# Vaccination Control in a Stochastic SVIR Epidemic Model

**DOI:** 10.1155/2015/271654

**Published:** 2015-05-24

**Authors:** Peter J. Witbooi, Grant E. Muller, Garth J. Van Schalkwyk

**Affiliations:** University of the Western Cape, Private Bag X17, Bellville 7535, South Africa

## Abstract

For a stochastic differential equation SVIR epidemic model with vaccination, we prove almost sure exponential stability of the disease-free equilibrium for *ℛ*_0_ < 1, where *ℛ*_0_ denotes the basic reproduction number of the underlying deterministic model. We study an optimal control problem for the stochastic model as well as for the underlying deterministic model. In order to solve the stochastic problem numerically, we use an approximation based on the solution of the deterministic model.

## 1. Introduction

The discovery of the first vaccine marked a major breakthrough in the battle against infectious diseases. The subsequent development of vaccines for various diseases has brought about remarkable results. Vaccination gained increasing popularity and success after it eradicated the smallpox outbreak of 1976 [1].

Vaccination is a commonly used method to control diseases such as measles, polio, and tuberculosis. Usually there are different schedules of dosage for different diseases and vaccines. For some diseases, doses should be taken by vaccinees several times and there must be some fixed time interval between two doses (see for instance Gabbuti et al. [2]). In a given population, the proportion of susceptibles who goes on to vaccination depends on different factors, one of which is the availability of the necessary resources.

There are numerous examples of vaccination models in the literature; see, for instance, the book of Brauer and Castillo-Chávez [3] or the journal papers [4–10]. With vaccination models one would be interested in the extent to which a vaccination program would reduce the basic reproduction number or how one can optimally roll out a vaccination program over time, in order to reach a certain target. In the latter case, optimal control theory is the obvious candidate to employ in the analysis [11–14]. It is usually the proportion of susceptibles who are admitted into vaccination, which is used as the control variable.

As a way of accommodating randomness in a compartmental epidemic model, several authors have proposed models with stochastic perturbation. This means that one modifies a system of ordinary differential equations (ODEs) by adding stochastic noise or stochastic perturbations, giving rise to a system of stochastic differential equations (SDEs) [7, 8, 15–18]. We shall refer to the original system of ODEs as the* underlying deterministic model*.

It is known (see the book of Mao [19], for instance) that the stability of a system can be improved by adding stochastic perturbations. SDE epidemic models have been studied by various authors until it was proved in research articles [9, 15, 20, 21] that stochastic perturbation improves stability of the disease-free equilibrium in the given models. SDE models with vaccination have been studied by Tornatore et al. [7, 8]. In particular in [8] it is shown that the model permits solutions that are almost surely positive, and a theorem on exponential stability in mean square stability is proved. The model of Tornatore et al. [8] will be further analyzed in this paper.

For SDE models in epidemiology, optimal control has not been studied (or at least not published) extensively. One of the reasons for this could very well be the difficulty with high dimensionality of the resulting partial differential equation (PDE) for the value function; see the paper of Sulem and Tapiero [22], for instance. A four-compartmental SIVR model such as in [7, 8] could easily lead to a PDE having the time variable together with three state variables. In this contribution we study the exponential stability of the stochastic model of Tornatore et al. [8] and control of vaccination both in the underlying deterministic model and in the stochastic model. In control problems, our goal is to characterize the vaccination rate (as control variable) which, on a finite time interval, minimizes the number of infected individuals balanced against the cost of vaccination. In the stochastic case we minimize an expected value. We use standard methods for the deterministic case and the Hamilton-Jacobi-Bellman equation in the stochastic case.

In Section 2 we study exponential stability of the disease-free equilibrium of the stochastic SIVR model that was introduced in [8]. We prove that almost sure exponential stability of the disease-free equilibrium prevails whenever *ℛ*_0_ < 1, *ℛ*_0_ being the basic reproduction number of the underlying deterministic model. The deterministic control problem is treated in Section 3, and we find a numerical solution for the optimal control problem by using the fourth-order Runge-Kutta method. The optimal control of the SDE model is presented in Section 4. We observe a similarity in the form of the control in the two cases, stochastic versus deterministic. On this basis, in Section 5 we propose that the numerical solution of the control problem of the underlying deterministic model can be used to compute an approximate solution to the stochastic control problem, assuming the perturbation parameter to be small. We present computational examples to illustrate our findings.

## 2. The Stochastic Model and Stability

In this section we introduce the SVIR model and we analyse the stability of the disease-free equilibrium. Firstly, we formulate the necessary assumptions for modeling with SDEs. Let us assume having a filtered complete probability space (*Ω*, *ℱ*, {*ℱ*_*t*_}_*t*≥*t*_0__, *ℙ*) and let *W*(*t*) be a one-dimensional Wiener process defined on this probability space.

We consider a stochastic SVIR model similar to the stochastic SIVR model of [8]. As in [8], the population is subdivided into four compartments/classes. These classes consist of all the individuals who are susceptible to the disease (*S*), under vaccination (*V*), infected with the disease (*I*), and removed (*R*).

It is assumed that births and deaths occur at the same constant rate *μ* and that all newborns enter the susceptible class. New infections occur at a rate *βSI*, for a constant *β* which is called the* contact rate*. A fraction *α*(*t*) of the susceptible class is being vaccinated at time *t*. The vaccination may reduce but not completely eliminate susceptibility to the disease. Therefore the model includes a factor *ρ* in the contact rate of vaccinated members, such that 0 ≤ *ρ* ≤ 1. If *ρ* = 0 the vaccination is perfectly effective while *ρ* = 1 means the vaccination has no effect at all. Furthermore, immunity to the disease is assumed to be permanent so that a fraction *γ* of infectives goes into the removed class.

The total population is constant and the variables are normalized so that

(1) $$\begin{array}{c} S\left(\;t\;\right)+V\left(\;t\;\right)+I\left(\;t\;\right)+R\left(\;t\;\right)=1\;\forall t\geq 0. \end{array}$$

The stochastic SVIR model is given as follows:

(2) dSt=μ−μSt−βItSt−αtStdt−σStItdWt,dVt=αtSt−ρβVtIt−μVtdt−ρσVtItdWt,dIt=βStIt+ρβVtIt−γIt−μItdt+σSt+ρVtItdWt,dRt=γIt−μRtdt.

Of course the parameters *μ*, *γ*, and *β* are positive constants. The parameter *σ* determines the intensity of the stochastic perturbation. If *σ* = 0, then the model reduces to the underlying deterministic model.

If *α* is constant then the model above is identical to that in [8], and the point

(3) E0=S0,I0,V0,R0=m,0,1−m,0with  m=μμ+α

is the disease-free equilibrium point of the underlying deterministic model and is the only equilibrium point of the stochastic model.

For some *n* ∈ *ℕ*, some *x*_0_ ∈ *ℝ* ^*n*^, and an *n*-dimensional Wiener process *B*(*t*), consider the general *n*-dimensional stochastic differential equation

(4) dxt=Fxt,tdt+Gxt,tdBt,x0=x0.

A solution to the above equation is denoted by *x*(*t*, *x*_0_). We assume that *F*(*t*, 0) = *G*(*t*, 0) = 0 for all *t* ≥ 0, so that the origin point is an equilibrium of (4).

**Definition 1 .**

The equilibrium *x* = 0 of the system equation (4) is said to be* almost surely exponentially stable* if, for all *x*_0_ ∈ *ℝ* ^*n*^,

(5) $$\begin{array}{c} \underset{t\to \infty }{\lim}\sup\frac{1}{t}\ln\left|\;x\left(t,x_{0}\right)\;\right|<0\;\text{a.s.} \end{array}$$

Let us denote by *ℒ* the differential operator associated with the function displayed in (4), defined for a function *U*(*t*, *x*) ∈ *C* ^1,2^(*ℝ* × *ℝ* ^*n*^) by

(6) $$\begin{array}{c} LU=\frac{\partial U}{\partial t}+F^{\text{trp}}\frac{\partial U}{\partial x}+\frac{1}{2}\text{Trc}\left[\;G^{\text{trp}}\frac{\partial^{2}U}{\partial x^{2}}G\;\right]. \end{array}$$

Here Trc means trace and trp denotes the transpose of a matrix.

For the remainder of this section we shall study stability and for this purpose we regard *α*(*t*) to be a positive constant, *α*(*t*) = *α* for all *t* > 0. In [8] it was shown that the system of SDEs has unique solutions which are almost surely positive. Thus we shall restrict ourselves to sample paths *w* ∈ *Ω* for which the coordinates are positive for all *t* ≥ 0. The following observation is towards the proof of the stability theorem. Recall that we have introduced the number *m* = *μ*(*μ* + *α*)^−1^ in expression (3). Let us define, for any constants *a* > 0 and *c* > 0, the stochastic processes:

(7) ztaSt−m2+It+cRt,u1t=−2aSt−mσStItzt,u2t=σSt+ρVtItzt.

Note that *I*(*t*)/*z*(*t*) ≤ 1 and *S*(*t*) + *ρV*(*t*) ≤ *S*(*t*) + *V*(*t*) ≤ 1. Now we calculate

(8) ∫0tu12sds∫0t4a2Ss−m2σ2S2sds≤4a2σ2t,∫0tu22sds∫0tσ2Ss+ρVs2ds≤σ2t.

Therefore by the strong law of large numbers for martingales (see [19]) it follows that

(9) $$\begin{array}{c} \underset{t\to \infty }{\lim}\sup\frac{1}{t}\overset{t}{\underset{0}{\int }}\left[\;u_{1}\left(s\right)+u_{2}\left(s\right)\;\right]dW\left(s\right)=0. \end{array}$$

**Proposition 2 .**

For constants *a* > 0 and *c* > 0, let *z*(*t*) = *a*(*S*(*t*) − *m*)^2^ + *I*(*t*) + *cR*(*t*), and let *U*(*t*) = ln⁡*z*(*t*). Then *z*(*t*) almost surely (a.s.) converges exponentially to 0 if

(10) $$\begin{array}{c} \underset{t\to \infty }{\lim}\sup LU\left(\;t\;\right)<0\;\left(a.s.\right). \end{array}$$

**Proof**

Using the Itô formula and with *u*_1_(*t*) and *u*_2_(*t*) defined as above, we can express *U*(*t*) as

(11) Ut=U0+∫0tLUsds+∫0tu1s+u2sdWs.

We have shown that

(12) $$\begin{array}{c} \underset{t\to \infty }{\lim}\sup\frac{1}{t}\overset{t}{\underset{0}{\int }}\left(\;u_{1}\left(s\right)+u_{2}\left(s\right)\;\right)dW\left(s\right)=0\;\left(\text{a.s.}\right), \end{array}$$

and hence the claim of the proposition follows readily.

The following observation from [9] is useful in exponential stability analysis.

**Lemma 3 .**

For *k* ∈ *ℕ*, let *X*(*t*) = (*X*_1_(*t*), *X*_2_(*t*),…, *X*_*k*_(*t*)) be a bounded *ℝ* ^*k*^-valued function. Let (*t*_0,*n*_) be any increasing unbounded sequence of positive real numbers. Then there is a family of sequences (*t*_*l*,*n*_) such that, for each *l* ∈ {1,2,…, *k*}, (*t*_*l*,*n*_) is a subsequence of (*t*_*l*−1,*n*_) and the sequence *X*_*l*_(*t*_*l*,*n*_) converges to the largest limit point of the sequence *X*_*l*_(*t*_*l*−1,*n*_).

Now we present our stability theorem. Recall that *ℛ*_0_ is the basic reproduction number of the underlying deterministic model, and *ℛ*_0_ = *β*/(*γ* + *μ*). Also recall from expression (3) that *m* = *μ*(*μ* + *α*)^−1^.

**Theorem 4 .**

If *ℛ*_0_ < 1, then the disease-free equilibrium point (*S*_0_, *I*_0_, *V*_0_, *R*_0_) = (*m*, 0,1 − *m*, 0) is almost surely exponentially stable.

**Proof**

The condition *ℛ*_0_ < 1 is equivalent to *β* − (*γ* + *μ*) < 0. Choose any number *c* > 0 such that *β* − (*γ* + *μ*) + *γc* < 0. Choose a number *a* > 0 such that

(13) $$\begin{array}{c} \beta -\left(\;\gamma +\mu \;\right)+\gamma c+a\left(\;2\beta +3\sigma^{2}\;\right)<0. \end{array}$$

Now let *z*(*t*) = *a*(*S*(*t*) − *m*)^2^ + *I*(*t*) + *cR*(*t*) and *U*(*t*) = ln⁡*z*(*t*). It suffices to prove that *z*(*t*) converges to 0 exponentially (a.s.). To this end, by Proposition 2 it suffices to prove that

(14) $$\begin{array}{c} \underset{t\to \infty }{\lim}\sup LU\left(\;t\;\right)<0\;\left(\text{a.s.}\right). \end{array}$$

Now we calculate *ℒU*(*t*). The latter can be expressed in the form

(15) $$\begin{array}{c} LU\left(\;t\;\right)=A\left(\;t\;\right)+B_{1}\left(\;t\;\right)+B_{2}\left(\;t\;\right)+B_{3}\left(\;t\;\right), \end{array}$$

with *A*(*t*), *B*_1_(*t*), *B*_2_(*t*), and *B*_3_(*t*) as follows:

(16) At=2am−Stztμ+αSt−m+βItSt+ItztβSt+ρβVt−γ−μ+cztγIt−μRt,B1t=2azt−4a2St−m22z2tσStIt2,B2t=−2aSt−mz2tσSt+ρVtItσStIt,B3t=−12z2tσSt+ρVtIt2.

With reference to *A*(*t*) we note that since *ρ* ≤ 1, we have

(17) βSt+ρβVtβSt+ρVt≤βSt+Vt≤β,

and therefore we obtain the inequality:

(18) At≤2am−Stztμ+αSt−m+βItSt+Itztβ−γ−μ+cztγIt−μRt.

In view of Lemma 3 we can define the following limits for a suitable increasing, unbounded sequence (*t*_*n*_):

(19) q=limn→∞⁡Stn−m2ztn,i=limn→∞⁡Itnztn,r=limn→∞⁡Rtnztn,s=limn→∞⁡Stn,v=limn→∞⁡Vtn,

and with

(20) $$\begin{array}{c} \underset{t\to \infty }{\mathrm{limsup}}LU\left(\;t\;\right)=\underset{n\to \infty }{\lim}LU\left(\;t_{n}\;\right). \end{array}$$

In particular then we have

(21) $$\begin{array}{c} aq+i+cr=1,\;0\leq s\leq 1,\;\;0\leq v\leq 1. \end{array}$$

Let

(22) $$\begin{array}{c} \Lambda =\underset{n\to \infty }{\lim}LU\left(\;t_{n}\;\right). \end{array}$$

We find an upper bound for *B*_1_(*t*) + *B*_2_(*t*) + *B*_3_(*t*). Since *S* ^2^ *I* ≤ 1 we have

(23) B1t122aztz2tσStIt2≤aσ2Itzt,B2t2aσ2Itzt2

since |*S*(*t*) − *m* | < 1. Noting that *B*_3_(*t*) ≤ 0 and *i* < 1, we obtain

(24) $$\begin{array}{c} \underset{n\to \infty }{\lim}B_{1}\left(\;t_{n}\;\right)+B_{2}\left(\;t_{n}\;\right)+B_{3}\left(\;t_{n}\;\right)\leq 3\sigma^{2}ai. \end{array}$$

Therefore Λ satisfies the following inequality:

(25) Λ≤−2μ+αaq+i−2aβss−m+βs+ρβv−γ+μ+cγi−cμr+3aσ2i.

Now we note that |2*aβs*(*s* − *m*)| ≤ 2*aβ*, and so we can write

(26) Λ≤−2μ+αaq+iβ−γ+μ+γc+2aβ+3aσ2−rcμ.

The coefficients of *q*, *i*, and *r* are negative (see (13)). Furthermore, *q*, *i*, and *r* cannot all be zero since

(27) $$\begin{array}{c} aq+i+cr=1. \end{array}$$

Therefore Λ < 0, and the proof is complete.

We run two sets of simulations of the deterministic and stochastic *S* and *I*-trajectories over *T* = 300 years. In the simulations, we use small perturbation parameters and small values of *V*. All parameter values in the computations except *β* and *γ* are the same in both scenarios and we also consider the same initial conditions. The common parameter values used in the computations are

 
*μ* = 0.016, *ρ* = 0.1, *α* = 0.3 and *σ* = 0.65

while the initial conditions are

 
*S*_0_ = 0.7, *V*_0_ = 0.05 and *I*_0_ = 0.15.

**Figure 1:**
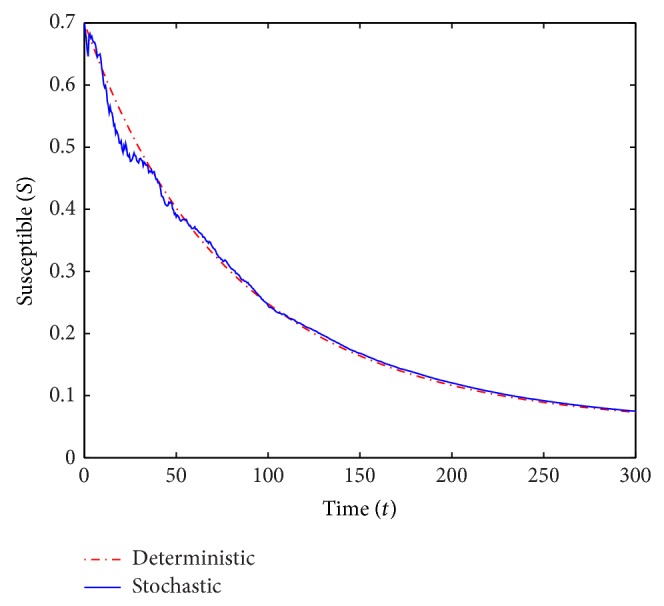
A simulation of *S* for the deterministic and stochastic models with *ℛ*_0_ = 0.893.

**Figure 2:**
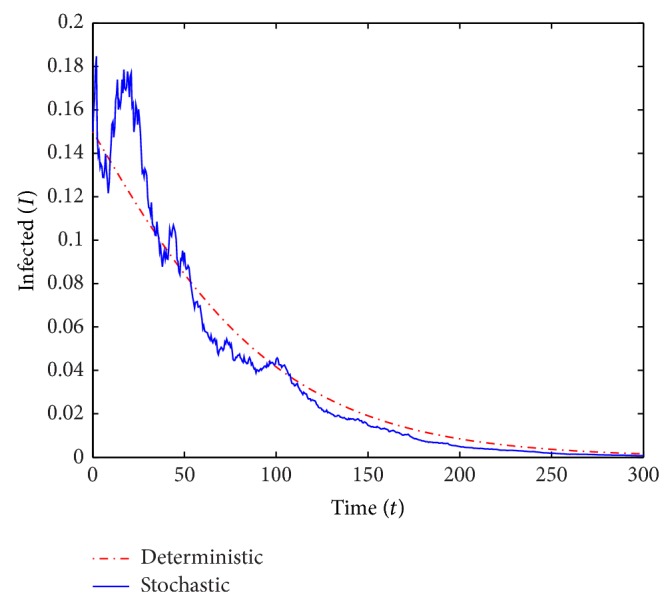
A simulation of *I* for the deterministic and stochastic models with *ℛ*_0_ = 0.893.

**Figure 3:**
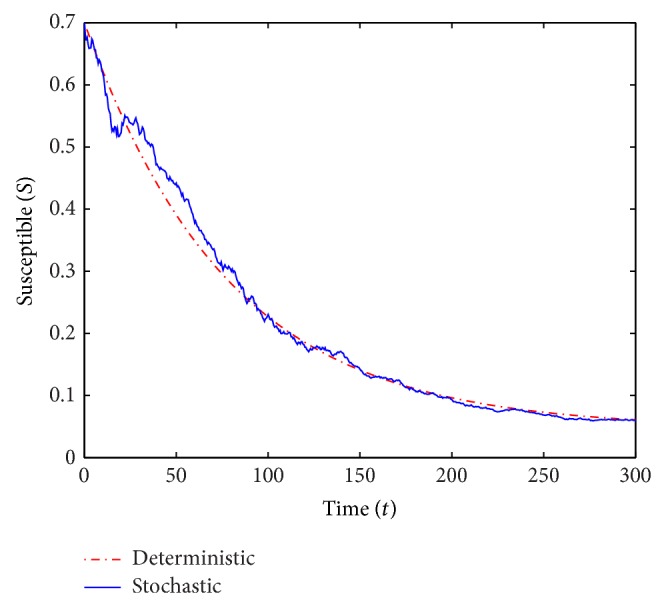
A simulation of *S* for the deterministic and stochastic models with *ℛ*_0_ = 1.724.

**Figure 4:**
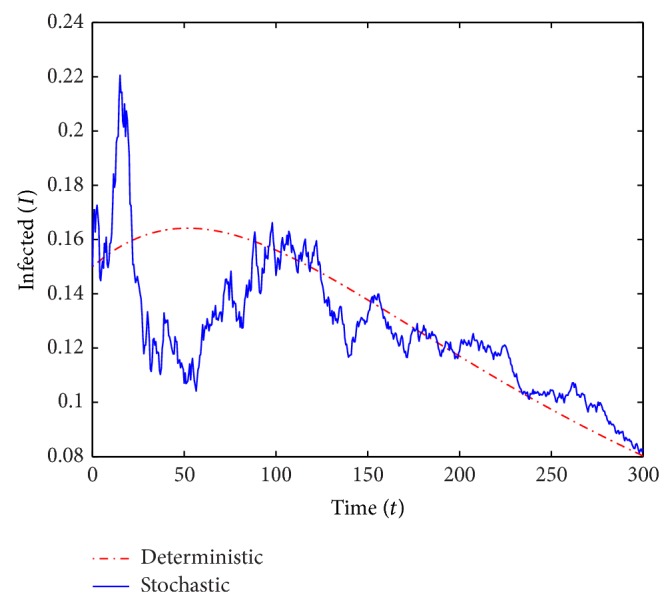
A simulation of *I* for the deterministic and stochastic models with *ℛ*_0_ = 1.724.

In the first run (see Figures 1 and 2) we use *β* = 0.55 and *γ* = 0.6, and we obtain *ℛ*_0_ = 0.893 < 1. In the second run (see Figures 3 and 4) we choose *β* = 0.2 and *γ* = 0.1, which yields *ℛ*_0_ = 1.724 > 1.

For *ℛ*_0_ < 1, Theorem 4 guarantees the disease-free equilibrium to be almost surely exponentially stable and indeed the *I*-curves in Figure 2 appear to converge to 0. For *ℛ*_0_ > 1 the theorem did not predict almost sure exponential stability and in fact the *I*-curves in Figure 4 (and many simulations with *ℛ*_0_ = 1.724 not shown) certainly do not show any clear intention of converging to 0.

## 3. The Deterministic Optimal Control Problem

We now formulate and solve the deterministic version of the control problem. Recall from Section 2 that in our SVIR model, *α*(*t*) represents the fraction of the susceptible class being vaccinated at time *t*. We wish to design an optimal vaccination schedule, *α* ^*∗*^(*t*), which minimizes a combination of the number of infectives on the one hand and the overall cost of the vaccination on the other hand, over a certain time horizon [0, *T*]. The cost of the vaccination is assumed to be proportional to the square of *α*(*t*).

For the purposes of optimization we introduce the functions *f*_1_(*t*), *f*_2_(*t*), and *f*_3_(*t*) appearing in the SDE system (2) as follows:

(28) f1tμ−μSt−βItSt−αtSt,f2t=αtSt−ρβVtIt−μVt,f3t=βStIt+ρβVtIt−γIt−μIt.

Now we can formulate the optimization problem.

**Problem 5 .**

Minimize the objective function

(29) $$\begin{array}{c} D\left(\;\alpha \left(\;\cdot \;\right)\;\right)=\overset{T}{\underset{0}{\int }}\left(\;\alpha^{2}\left(\;t\;\right)+cI\left(\;t\;\right)\;\right)dt \end{array}$$

subject to

 
$\dot{S}(t)=f_{1}(t)$, $\dot{V}(t)=f_{2}(t)$, $\dot{I}(t)=f_{3}(t)$,
 
*S*(0) = *S*_0_ ≥ 0, *V*(0) = *V*_0_ ≥ 0, *I*(0) = *I*_0_ ≥ 0.

The control variable *α*(*t*) is assumed to be a measurable function of time and bounded: $0\leq \alpha (t)\leq \bar{\alpha }\leq 1$.

We solve Problem 5 using well-established control theory such as in the book [23] of Lenhart and Workman. We construct the Hamiltonian function, and to this end we introduce Lagrange multipliers *λ*_1_(*t*), *λ*_2_(*t*), and *λ*_3_(*t*), also referred to as the costate variables. In the theorem below, the control variable, the state variables, and the costate variables are functions of time, but this dependence is suppressed except where required explicitly. The Hamiltonian of Problem 5 has the form

(30) Ht,S,V,I,αt=α2+cI+λ1f1t,S,V,I,α+λ2f2t,S,V,I,α+λ3f3t,S,V,I,α.

**Theorem 6 .**

An optimal solution for Problem 5 exists and satisfies the following system of differentiable equations:

(31) λ1˙λ1μ+βI+α−λ2α−λ3βI,λ2˙=λ2ρβI+μ−λ3ρβI,λ3˙=−c+λ1βS+λ2ρβV−λ3βS+ρβV−γ−μ,

with transversality conditions *λ*_1_(*T*) = 0, *λ*_2_(*T*) = 0, and *λ*_3_(*T*) = 0. Furthermore, the optimal vaccination rate is given by

(32) $$\begin{array}{c} \alpha^{*}\left(\;t\;\right)=\min\left[\;\max\left(\;0,\frac{1}{2}S\left(t\right)\left[\lambda_{1}\left(t\right)-\lambda_{2}\left(t\right)\right]\;\right),\bar{\alpha }\;\right]. \end{array}$$

**Proof**

In particular the Hamiltonian is convex with respect to *α*(*t*) and the existence of a solution follows. We check the first-order conditions for this optimization problem. We calculate the partial derivatives of the Hamiltonian with respect to the different state variables to obtain the time derivatives $\dot{\lambda_{i}}(t)$ of the costate variables. The calculation of

 
$\dot{\lambda_{1}}(t)=-\partial H/\partial S$, $\dot{\lambda_{2}}(t)=-\partial H/\partial V$, and $\dot{\lambda_{3}}(t)=-\partial H/\partial I$

leads to the equations asserted in the theorem. We now turn to the final part of the proof, which is about the form of the optimal control, *α* ^*∗*^(*t*). Since the function *α* ^*∗*^(*t*) must optimize the Hamiltonian, we calculate

(33) $$\begin{array}{c} \frac{\partial H}{\partial \alpha }=2\alpha -S\left(\;\lambda_{1}-\lambda_{2}\;\right). \end{array}$$

Consider now a fixed value of *t*. If 2*α* − *S*(*λ*_1_ − *λ*_2_) is zero for some value *α*(*t*) in the interval $\left[0,\bar{\alpha }\right]$, then the given value of *α*(*t*) is optimal. If for every $\bar{\bar{\alpha }}\in [0,\bar{\alpha }]$ we have $2\bar{\bar{\alpha }}-S(\lambda_{1}-\lambda_{2})\geq 0$ (resp., $2\bar{\bar{\alpha }}-S(\lambda_{1}-\lambda_{2})\leq 0$), then we must choose *α*(*t*) = 0 (resp., $\alpha (t)=\bar{\alpha }$). Thus we must have

(34) $$\begin{array}{c} \alpha^{*}\left(\;t\;\right)=\min\left[\;\max\left(\;0,\frac{1}{2}S\left(t\right)\left[\lambda_{1}\left(t\right)-\lambda_{2}\left(t\right)\right]\;\right),\bar{\alpha }\;\right]. \end{array}$$

**Figure 5:**
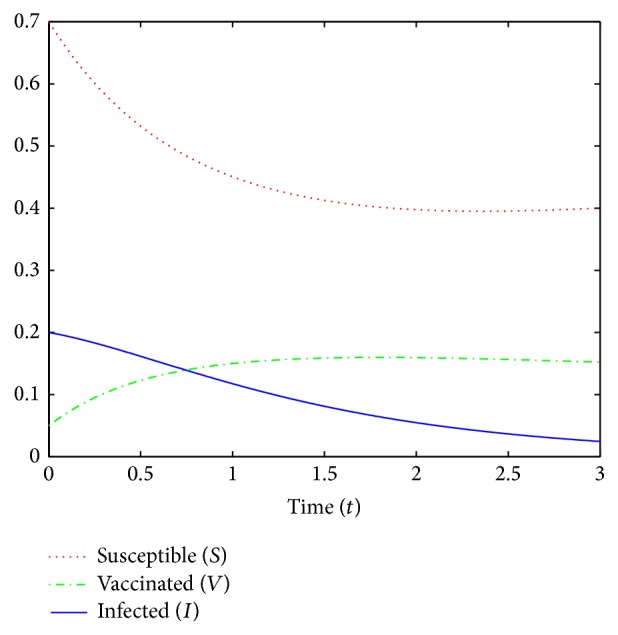
A simulation of *S*, *V*, and *I* for the deterministic model subject to the optimal control *α* ^*∗*^.

**Figure 6:**
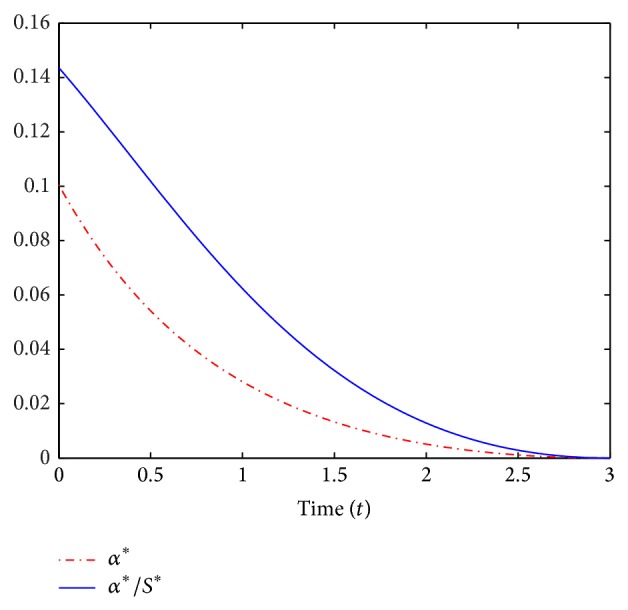
A simulation of *α* ^*∗*^ and *α* ^*∗*^/*S* ^*∗*^ for the deterministic model.

In Figure 5 we plot for *T* = 3 years the *S*, *V*, and *I*-trajectories of the deterministic model subject to the optimal control *α* ^*∗*^. We simulate *α* ^*∗*^ and *α* ^*∗*^/*S* ^*∗*^ for the deterministic model in Figure 6. In the simulations, we consider the parameters *c* = 0.3, *μ* = 0.016, *β* = 0.55, *ρ* = 0.1, *γ* = 0.45, and $\bar{\alpha }=0.8$ and initial conditions *S*_0_ = 0.7, *V*_0_ = 0.05, and *I*_0_ = 0.2.

In Figure 6 the dashed curve represents *α* ^*∗*^(*t*), that is, the optimal vaccination rate. In this illustration, the optimal vaccination roll-out starts with initial vaccination of approximately 0.11 and decreases gradually over the next 3 years.

## 4. The Stochastic Optimal Control Problem

In this section we formulate the stochastic version of the optimization problem and describe its solution. For stochastic control theory we refer to the book [24] of Øksendal. Our objective is to find an optimal vaccination rate *α* ^*∗*^(*t*) that minimizes the objective functional which for an initial state *x*_0_ is defined by

(35) $$\begin{array}{c} E_{0,x_{0}}\left[\;\overset{T}{\underset{0}{\int }}\left(\;\alpha^{2}\left(\;s\;\right)+cI\left(\;s\;\right)\;\right)ds\;\right]. \end{array}$$

Here the expectation is obtained on the condition that the initial state (at time *t* = 0) of the system is *x*_0_. In step with the deterministic problem of earlier, we assume that there is a fixed constant $\bar{\alpha }\leq 1$ with $\alpha (t)\leq \bar{\alpha }$ (a.s.). The class of admissible control laws is

(36) $$\begin{array}{c} A=\left\{\;\alpha \left(\;\cdot \;\right):\alpha \;\;\text{is adapted},\text{and}\;\;0\leq \alpha \leq \bar{\alpha }\;\;\text{a.s.}\;\right\}. \end{array}$$

To solve this stochastic control problem, we define the performance criterion as follows:

(37) $$\begin{array}{c} J\left(\;t,x;\alpha \;\right)=E_{t,x}\left[\;\overset{T}{\underset{t}{\int }}\left(\;\alpha^{2}\left(\;s\;\right)+cI\left(\;s\;\right)\;\right)ds\;\right], \end{array}$$

where the expectation is conditional on the state of the system being a fixed value *x* at time *t*. We define the value function as

(38) $$\begin{array}{c} U\left(\;t,x\;\right)=\underset{\alpha \left(\cdot \right)\in A}{\inf}J\left(\;t,x;\alpha \;\right)=J\left(\;t,x;\alpha^{*}\;\right). \end{array}$$

We determine a control law that minimizes the expected value *J* : *𝒜* → *ℝ*_+_ given by (37). We now formulate the stochastic analogue of the optimal control problem, subsequent to which we present the solution formulae.

**Problem 7 .**

Given the system (4) and given *𝒜* as in (36) with *J* as in (37), find the value function

(39) $$\begin{array}{c} U\left(\;t,x\;\right)=\underset{\alpha \in A}{\inf}J\left(\;t,x;\alpha \;\right), \end{array}$$

and an optimal control function

(40) $$\begin{array}{c} \alpha^{*}\left(\;t\;\right)=\arg\underset{\alpha \in A}{\inf}J\left(\;x;\alpha \left(t\right)\;\right)\in A. \end{array}$$

We can find an expression for the optimal vaccination strategy *α* ^*∗*^ through the following theorem.

**Theorem 8 .**

A solution to the optimal vaccination problem stated in Problem (36) is of the form

(41) $$\begin{array}{c} \alpha^{*}\left(\;t\;\right)=\min\left[\;\max\left(\;0,\frac{1}{2}S\left(t\right)\left[U_{S}\left(t\right)-U_{V}\left(t\right)\right]\;\right),\bar{\alpha }\;\right]. \end{array}$$

**Proof**

We determine (41) via the dynamic programming approach. First we calculate *ℒU*(*t*):

(42) LUt=f1tUSt+f2tUVt+f3tUIt+12σStIt2USSt+12ρσStIt2UVVt+12σSt+ρVtIt2UIIt+ρσIt2StVtUSVt−σIt2StSt+ρVtUSIt−ρσIt2VtSt+ρVtUVIt.

Applying the Hamilton-Jacobi-Bellman theory (see, for instance, [24]) we must find the infimum:

(43) $$\begin{array}{c} \underset{\alpha \in A}{\inf}\left[\;\alpha^{2}\left(\;t\;\right)+cI\left(\;t\;\right)+LU\left(\;t\;\right)\;\right]. \end{array}$$

For this purpose, we need to find the partial derivative of the expression

(44) $$\begin{array}{c} \alpha^{2}\left(\;t\;\right)+cI\left(\;t\;\right)+LU\left(\;t\;\right) \end{array}$$

with respect to *α*, and this derivative should vanish. This leads to the equation:

(45) $$\begin{array}{c} 2\alpha \left(\;t\;\right)-S\left(\;t\;\right)U_{S}\left(\;t\;\right)+S\left(\;t\;\right)U_{V}\left(\;t\;\right)=0. \end{array}$$

We consider the bounds on *α* and by an argument similar to that in the proof of the deterministic case; the asserted expression for *α* ^*∗*^(*t*) emerges.

## 5. Numerical Example

In the discussion that follows, the computations are done for *T* = 3 years and we consider three different values of the perturbation parameter, *σ*. In particular we consider

 the *σ*-values: *σ* ∈ {0.15,0.30,0.45}

together with the following parameter values and initial conditions:

 
*c* = 0.3, *μ* = 0.016, *β* = 0.55, *ρ* = 0.1, *γ* = 0.45, and $\bar{\alpha }=0.8$,
 
*S*_0_ = 0.7, *V*_0_ = 0.05, and *I*_0_ = 0.2.

For each value of *σ* above, we use the results of the deterministic control problem to find an approximate numerical solution for the stochastic control problem. In particular, we use *λ*_1_ − *λ*_2_ as a proxy for *U*_*S*_ − *U*_*V*_ in the calculation of *α* ^*∗*^ in this case. We note that the presence of *S*(*t*) makes *U* into a stochastic variable even with the said proxy (in the stochastic case).

For each value of *σ*, we show in Table 1 the *U*(0)-values obtained as the average over 3000 runs made for different candidates for $\hat{\alpha }$ as we take $\alpha =S\hat{\alpha }$. In these runs we take *λ*_1_ and *λ*_2_ of the underlying deterministic model.

Using a contact rate *β*_0_ = *β* − (*σ* ^2^/2) instead of *β*, we choose the first candidate

(46) $$\begin{array}{c} {\hat{\alpha }}_{0}\left(\;t\;\right)=\frac{1}{2}\left[\;\lambda_{1}\left(\;t\;\right)-\lambda_{2}\left(\;t\;\right)\;\right]. \end{array}$$

The adjustment on the contact rate is motivated by the general stabilizing effect of this type of stochastic perturbation and the perturbation being associated with the parameter *β*.

We also take

(47) $$\begin{array}{c} {\hat{\alpha }}_{i}\left(\;t\;\right)={\hat{\alpha }}_{0}\left(\;t\;\right)+\epsilon i-\frac{\epsilon i}{3}t,\;\text{for}\;\;i=-3,-2,-1,1,2,3 \end{array}$$

corresponding to the rate *β* and with *ϵ* = 0.001.

Furthermore, we consider

(48) $$\begin{array}{c} {\hat{\alpha }}_{4}\left(\;t\;\right)=\frac{1}{2}\left[\;\lambda_{1}\left(\;t\;\right)-\lambda_{2}\left(\;t\;\right)\;\right], \end{array}$$

corresponding to the contact rate *β*.

Just for further comparison we choose a linear curve candidate ${\hat{\alpha }}_{5}(t)$, say,

(49) $$\begin{array}{c} {\hat{\alpha }}_{5}\left(\;t\;\right)=\eta +\varphi t \end{array}$$

with *η* = 0.0001 and *φ* = 0.00015, also corresponding to a contact rate of *β*.

Let us use the notation *U*_*i*_(0) for the value of *U*(0) corresponding to the control $S{\hat{\alpha }}_{i}$. We notice that *U*_4_(0) and *U*_5_(0) are bigger than *U*_0_(0). Even more interesting is the pattern *U*_−3_(0) > *U*_−2_(0) > *U*_−1_(0) > *U*_0_(0) and *U*_0_(0) < *U*_1_(0) < *U*_2_(0) < *U*_3_(0). This creates the impression that *U*_0_(0) is close to being a minimum. At least, with all the difficulties of a more precise solution, the choice of ${\hat{\alpha }}_{0}$ seems like a viable option in a real application. The inequalities  *U*_−3_(0) > *U*_−2_(0) > *U*_−1_(0) > *U*_0_(0) and *U*_0_(0) < *U*_1_(0) < *U*_2_(0) < *U*_3_(0) has been tested for other parameter choices too, with *σ* small. The simulations tested all revealed the same behaviour.

**Figure 7:**
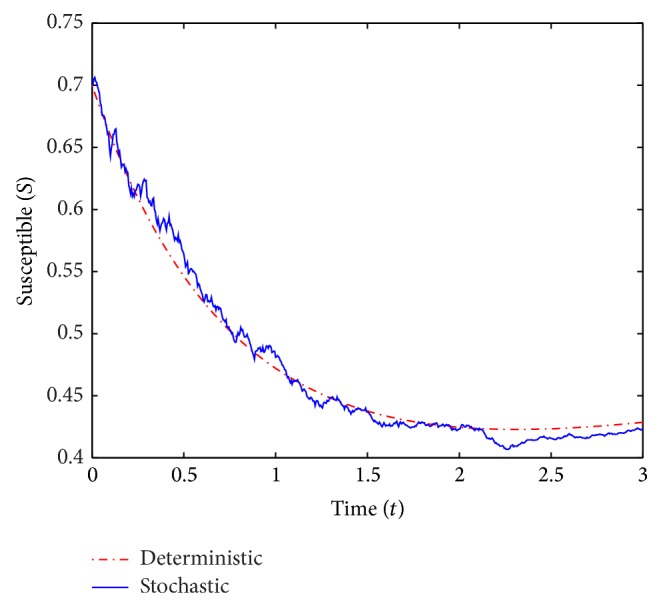
A simulation of *S* ^*∗*^(*t*) for the deterministic and stochastic models.

**Figure 8:**
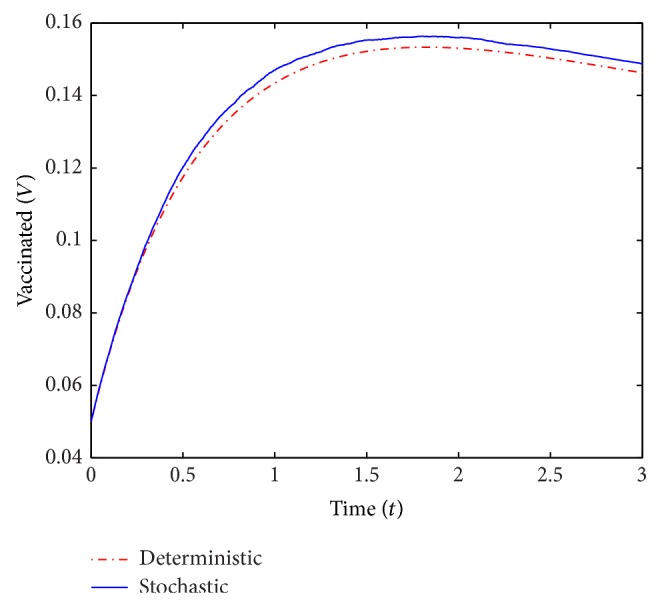
A simulation of *V* ^*∗*^(*t*) for the deterministic and stochastic models.

**Figure 9:**
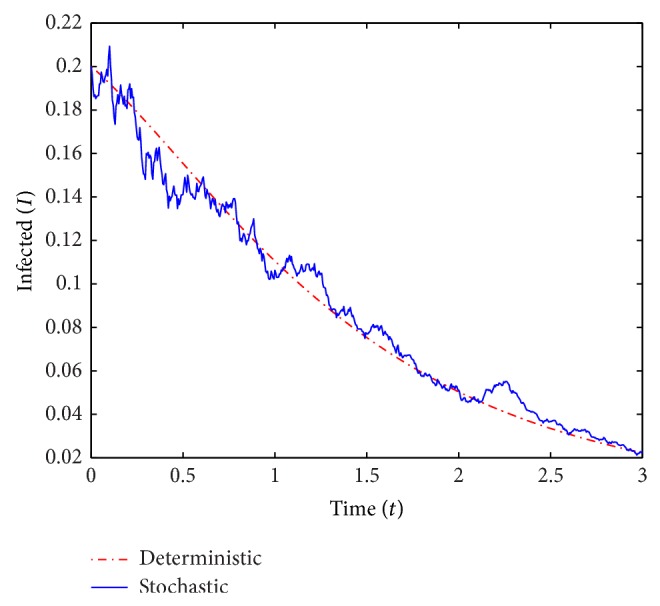
A simulation of *I* ^*∗*^(*t*) for the deterministic and stochastic models.

In Figures 7–9 we simulate the optimal solutions *S* ^*∗*^, *V* ^*∗*^, and *I* ^*∗*^ of the deterministic and stochastic (*σ* = 0.3) models. We use the same values for the parameters *c*, *μ*, *β*, *ρ*, *γ*, *σ*, $\bar{\alpha }$, and initial conditions *S*_0_, *V*_0_, and *I*_0_ as in the discussion preceding these simulations.

An important point to note about our approximation is that it fully accommodates the stochasticity (embodied and concentrated in the factor *S* of the expression for *α* ^*∗*^(*t*)). Therefore it gives a very good approximation, at least in the sense that in Table 1, the minimum value of *U*(0) consistently corresponds to ${\hat{\alpha }}_{0}$.

## 6. Conclusion

This paper presents some further insights into a stochastic vaccination model introduced in the paper of Tornatore et al. [8]. Our investigations covered two important aspects: exponential stability of the disease-free equilibrium and optimal control of vaccination.

Regarding stability, the main result of our paper has a particularly simple formulation. Essentially it says that we have almost sure exponential stability whenever the basic reproduction number of the underlying deterministic model is below unity. It will be good to know how an increase in vaccination rate would perhaps lead to better stability of the stochastic model. Nevertheless, the result as it stands is a good assurance.

As for the control problem, on the basis of a popular type of objective functional, we designed an efficient strategy for the roll-out of vaccination. It roughly amounts to minimizing the infections, balanced against the cost of vaccination. We exploit a similarity between the forms of the optimal controls for the stochastic model and the underlying deterministic model; then we use the relative simplicity of the latter to find approximate numerical solutions for the stochastic optimal control. Numerical simulation enables us to assess the feasibility of the option we followed, for a specific example. A more formal approach to the numerical solution of the optimal control problem is far more intricate and labour-intensive, and our method is a workable alternative. This could be the starting point for more sophisticated approximation methods.

## Figures and Tables

**Table 1:** 

*U*(0) for different candidates of $\hat{\alpha }$ and *σ*			
${\hat{\alpha }}_{i}$	*σ* = 0.15	*σ* = 0.3	*σ* = 0.45
${\hat{\alpha }}_{-3}$	29.84672	34.22981	36.21729
${\hat{\alpha }}_{-2}$	29.26219	34.09944	36.15703
${\hat{\alpha }}_{-1}$	28.96307	34.07355	36.14670
${\hat{\alpha }}_{0}$	28.91028	33.93111	35.95781
${\hat{\alpha }}_{1}$	29.52337	34.34593	36.26951
${\hat{\alpha }}_{2}$	30.15975	34.56046	36.36480
${\hat{\alpha }}_{3}$	31.01890	34.80816	36.47420
${\hat{\alpha }}_{4}$	29.03129	34.07210	36.05649
${\hat{\alpha }}_{5}$	30.71396	35.24172	36.82271
